# Reminiscences of Hawaii—En Route for Captain Cook’s Monument

**Published:** 1913-05

**Authors:** John C. Warbrick

**Affiliations:** Chicago


### TEXAS MEDICAL JOURNAL.

Established July, 1885

F. E. DANIEL, M. D., - - - - Editor, Publisher and Proprietor

Published Monthly.—Subscription, $1.00 a Year.

VOL. XXIX. AUSTIN, MAY, 1913. No. 11.

The publisher is not responsible for the views of the contributors.

### Original Articles.

For Texas Medical Journal.

### Reminiscences of Hawaii—En Route for Captain Cook's Monument.

BY JOHN C. WARBRICK, M. D., CHICAGO.

Up early, at 5 o'clock, on G. W. McDougall's Coffee Plantation. we quickly mounted horses and it did not take us long to cover the distance of four miles down the mountain and get to Hailua, North Kona, Hawaii, where the inter-island passenger and freight steamer, the *W. G. Hall* was already off the port at 6 a. m., October 3rd, 1904, somewhat earlier than usual on account of having a good deal larger cargo to discharge. This is always sent ashore as has been the custom for years, in the small boats belonging to the vessel—the large steamers not being able to get in close enough to the shore to land freight and passengers, on account of the shallowness of the water.

The cargo mostly consisted of a variety of merchandise, such as bags of flour, provisions, etc., for the island people, but at rare times, an interesting event would happen, such as a horse having to be lowered from the boat into the water and be made to swim to land, to its owner, and the performance was gone through that morning. This is the only means they have of getting them to and from the boat, always anchored some distance out from the shore.

Now whenever it is necessary to ship cattle from Hailua to Honolulu, distance of more than one hundred miles, a novel method is used, that has been carried on for years. Each animal has to be lassoed by a man on horseback, who then, at full speed, dashes into the water, dragging it after him, beyond the animal's depth, when another man takes it and fastens it to the edge of a small boat backwards by means of its horns, thus the weight of it hanging downwards in the water, makes it impossible to get loose.

One by one the cattle are put on each side of the small boat in that way until six or seven are placed on each side, then it is gradually pulled through the water by a big rope to the steamer, where they are hauled, one by one, on board.

The freight for each place, was always landed as early as possible, and the moment the last boat was being hauled up the side of the steamer, the captain lost no time in getting away to the next stopping place.

That morning we got away in good time, and soon after the boat started down the coast, an agreeable surprise was to find on board a former traveling acquaintance named Baker.

He was on his way to get some experience on a sugar plantation, at a place called Pahalo, some distance down the coast, and inland, so went that far together. Baker soon got all the experience he wanted, for not long afterwards, meeting him again, he told me, life on a sugar plantation was not to his liking in any way. The hours (from five in the morning, until six at night) were too long and tiresome, while it was far more strenuous than he expected or could stand, so he gave it up, preferring something easier; for Baker was an Englishman, and not accustomed to the continual driving of men, often with epithets, hard labor and strict hours on a sugar plantation, that takes a toughened and well-seasoned cowboy to stand and maintain discipline at all times.

Sailing down the coast, there was nothing much to see for a time, except the foliage on the mountain, studded here and there by a house; however, we were soon to stop at a very interesting, historical spot, known the whole world over to sailors and mariners as the place where the famous navigator, Captain Cook, who discovered the islands, was killed, in the year 1778, by the natives; a monument being erected to his memory.

This place was Kealakekua Bay, a pretty spot, lying, as it does, snugly sheltered beneath the high, massive cliffs, covered with numerous trees on the land side, and ever open to the continual surging waves of the lonely ocean on the other.

As soon as the steamer was off the port, Baker suggested going ashore to see what there was; so we made the first boat lowered and soon got to land, in order to have as much time as possible to look around.

The village is a very small one, natives being about the only inhabitants; living as they do in huts or small board houses, simple, plain and quiet existence; also, rather dreary it would seem, but nevertheless they are happy and contented. They have never known anything different and much civilization would soon kill them off. A boat call is generally the most important and exciting event that ever happens, as the years roll on, to attract attention; remote and sequestered as it is far from the world's highway of commerce.

Possibly, very few people of the present generation hardly know that Captain Cook discovered the islands or that a monument was erected to his memory at Healakekua Bay, so fleeting is fame and so little does it concern most people, and in the lines so often quoted in reference to this:

Time like an ever-rolling stream,
Bears all its sons away.
They die, forgotten as a dream
Dies at the break of day.

The natives, anxious to make some money, freely offered to sell us cocoanuts at the low price of five for twenty-five cents. The native boys or Kanakas, as they are called, will soon climb up to the top of one of the tall slender trees, especially if any money is shown to them, and pulling the cocoanuts, drop them to the ground, whenever they need any for visitors.

As they are very nimble, they do this by putting their feet against the tree and their arms around it, and thus climb up without any fear or trouble, and sit on the top.

Baker bought two or three cocoanuts and took them on the boat, to make up some kind of a mixture with the milk of the nuts, which he thought was good to drink.

The monument erected to the memory of Captain Cook, is not far away from the place of landing and easy of access. It is on one side of the bay, close to the shore, or about fifteen yards from where he was stabbed to death; sheltered beneath the nearby cocoanut trees, and the huge over-hanging cliffs, a little farther away at the end of the bay. It is only a plain obelisk, about seven feet high, while a thin, narrow iron railing, a foot from the ground, is all that fences it in; while in some places, time is gradually causing it to crumble, and visitors take away whatever pieces of it they can get. It had the appearance of being forgotten generally; the way in time with most things, while from its condition, the natives can hardly give it much consideration at any time.

On the monument are the words: "In memory of Captain James Cook, who discovered the islands, January 18, 1778, and fell near the spot on February 14, 1778." The monument was erected in November, 1874, by his fellow countrymen, through Lord Byron, who visited the place in his vessel, which he commanded in 1825.

Captain Cook made three voyages to the islands and it was while he was on his third expedition, that he was stabbed to death by a native and his body burned, at the early age of 51 years.

